# Evaluation of a digital remote extraction analysis and monitoring tool for key performance indicators (KPIs) in the blood culture process

**DOI:** 10.1007/s10096-025-05238-x

**Published:** 2025-08-18

**Authors:** Claudio Palmieri, Laura Bartolini, Andrea Berlingeri, Barbara Camilloni, Gianfranco La Bella, Michela Pascarella, Marina Selleri, Felice Valzano, Chiara Vismara, Simone Ambretti, Carla Fontana, Mario Rassu, Gian Maria Rossolini, Fabio Arena, Antonella Mencacci

**Affiliations:** 1 Becton Dickinson Italia S.p.A, Milan, Italy; 2 Microbiology and Virology Unit, Careggi University Hospital, Florence, Italy; 3 Microbiology Unit, IRCCS Azienda Ospedaliero-Universitaria di Bologna, Bologna, Italy; 4 Department of Medicine and Surgery, Medical Microbiology Section, University of Perugia, Perugia, Italy; 5 Santa Maria della Misericordia Hospital, Perugia, Italy; 6 Department of Clinical and Experimental Medicine, University of Foggia, Foggia, Italy; 7 Azienda Sanitaria Locale della Provincia di Foggia, Foggia, Italy; 8 Department of Microbiology, San Bortolo Hospital, Vicenza, Italy; 9 National Institute for Infectious Diseases “L. Spallanzani” I.R.C.C.S, Rome, Italy; 10 Clinical Microbiology, ASST Grande Ospedale Metropolitano Niguarda, Milan, Italy; 11 Department of Medical and Surgical Sciences, University of Bologna, Bologna, Italy; 12 Department of Experimental and Clinical Medicine, University of Florence, Florence, Italy; 13 Microbiology and Virology Unit, Policlinico Foggia, Foggia, Italy

**Keywords:** Blood cultures, Bloodstream infections, Diagnostic microbiology, Key performance indicators

## Abstract

**Purpose:**

Bloodstream infections (BSIs) pose a significant health threat, requiring effective diagnostic processes to ensure appropriate treatment. Monitoring the quality of blood cultures (BCs) process (the cornerstone of BSIs etiological diagnosis) is critical. This study aimed to describe an automated tool, BD DREAM™ (Digital Remote Extraction Analysis & Monitoring), designed for monitoring key performance indicators (KPIs) in the BCs process.

**Methods:**

A multicenter study was conducted across six Italian hospitals, analyzing 234,978 BC bottles, associated with 55,819 episodes. The system monitored KPIs related to mean blood volume per bottle, rates of solitary episodes, rate of BCs from central venous catheter (CVC) unpaired with concomitant BCs from peripheral vein, and contamination rates.

**Results:**

The findings highlighted significant variability in BC practices among centers and hospital services, with mean blood volume per bottle below recommended standards (6.45 mL vs. ≥ 8.0 mL), with 21.8% (range 3.2–62.9%) of episodes classified as solitary. The rate of BCs from CVC unpaired with concomitant BCs from peripheral vein was 14.9% (range 10.2–39.0%). Overall, contamination rates ranged from 4.2 to 4.7%, depending on the criteria adopted for definition. The setting with the more critical values was medical area.

**Conclusion:**

The tool was useful for immediate visualization and identification of hospital services with critical performance. These results suggest that integrating BC digital monitoring tools into clinical microbiology workflows is possible. Further studies will be needed to demonstrate that the implementation of BD DREAM™ can lead to enhancement of diagnostic accuracy, optimization of resources allocation, and improvement of patient outcomes.

**Supplementary Information:**

The online version contains supplementary material available at 10.1007/s10096-025-05238-x.

## Introduction

Bloodstream infection (BSI) is defined as bacteremia with a “true” pathogen which can often be demonstrated by the positivity of one or more blood cultures. In the suspicion of an infection involving the bloodstream, it is mandatory to search for pathogens in the patient’s blood, and the diagnosis of BSIs represents one of the critical functions for the Clinical Microbiology Laboratory [1–3].

Faster identification of the causing microorganism and accurate susceptibility testing improve the care of patients with BSIs and leads to a tailored therapy which grows in importance in settings with high antimicrobial resistance rates [1, 4–7].

Despite the introduction of new technologies, blood culture (BC) remains the gold standard in the microbiological diagnosis of BSI/sepsis [8]. The results of BCs are, to a large extent, determined by the quality of the pre-analytical phase with skin disinfection, amount of blood per bottle, and number of bottles collected per episode being crucial for diagnosis [8–11].

Given the multifaceted range of actions needed to improve the microbiological diagnosis of BSI, continuous quality improvement initiatives are urgently needed. BCs improvement programs should be considered a hospital priority [12]. These approaches include the identification of relevant key performance indicators (KPIs) to monitor critical points in the BC process. These KPIs should be measured as baseline and be used to define the targets at hospital and unit level [12]– [13]. The possibility to regularly access these data to improve the quality of the BC process is of fundamental importance not only for the entire hospital, but especially for individual departments/units of the hospital. Unfortunately, due to the fragmentation of information and the nature of data, monitoring KPIs for BSIs is a time-consuming activity that is prohibitive for the majority of Clinical Microbiology Laboratories. For this reason, feasible approaches for robust and automated KPIs calculation are urgently needed.

In this study, we describe a standardized and automated tool for monitoring the main KPIs of the pre-analytical and analytical phases of the diagnostic pathway for BSI/sepsis. Furthermore, we provide an updated overview on these indicators in the clinical setting.

## Materials and methods

### Digital remote extraction analysis & monitoring tool

The informatic tool used in this study is a BD (BECTON DICKINSON ITALIA S.p.A., Milan, Italy) proprietary software pipeline, named BD DREAM™ (Digital Remote Extraction Analysis & Monitoring) BSI, created for automated KPIs calculation.

BD DREAM™ automatically processes data extracted from BD Epicenter ™, the platform that provides advanced data management for BD microbiology systems, including the BD BACTEC™ blood culture system, BD Phoenix™ automated identification and susceptibility test system (all from BECTON DICKINSON and Company, Franklin Lakes, New Jersey, US), and Bruker MALDI Biotyper^®^ (Bruker Daltonics GmbH & Co. KG, Bremen, Germany) and from the Laboratory Information System (LIS). All centers were equipped with a standard bi-directional interface between BD Epicenter™ and their respective LIS systems (Weblab” TESI GROUP, Milan, Italy; “TD Synergy” Siemens Healthcare s.r.l., Milan, Italy; “DNLAB” Dedalus, Santiago, Chile; ThemixLab, Thermix Italia, Rome, Italy).

Input data include: anonymized patient demographics (the EpiCenter Database De-identification Utility is used to ensure data anonymization), admission ward, numeric progressive sample identification code, bottle identification number, specimen origin (peripheral vein, sample from intravascular invasive devices such as central venous catheter), date, time, instrument test status (flag positive/negative), bottle type (anaerobic/aerobic), identification at the species level of bacterial/fungal isolates (performed by mass spectrometry) obtained from each bottle flagged positive. Blood volume was measured by the BVM EpiCenter tool. The BD DREAM™ tool calculates a weighted mean per center and per hospitalization area for the in-scope hospital services based on BVM report [14].

**Fig. 1:**
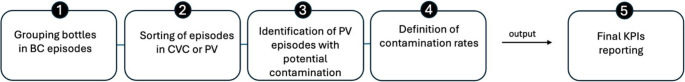
Schematic representation of the BD DREAM™ BSI process. (1) grouping of BC bottles collected within 24 h from a single patient in a given area; (2) sorting of episodes consisting exclusively of a group of BC bottles obtained from a peripheral vein and episodes consisting of a group of BC bottles with at least one sample from a central venous catheter; (3) identification of BCs positive for species regarded as possible contaminants; (4) definition of contamination rates; (5) KPIs reporting stratified at hospital or ward level. BC: blood culture; CVC central venous catheter; PV: peripheral vein

After input data extraction from both BD EpiCenter™ and center’s LIS, BD DREAM™ BSI proceeds with automated analysis following the steps illustrated in Fig. 1 adopting the standard definitions summarized in Table 1.

**Table 1:** Standard definitions adopted for the analysis

Definition	Explanation
BC set	Pair of BC bottles (aerobic and anaerobic), or single bottles, obtained from the same sampling procedure; applies for multi-sampling strategy only (MSS).
BC episode	Group of BC bottles collected within 24 h (after the first index BC sample that defines hour 0) from a single patient in a hospital area
PV episode	Episode consisting of a group of BC bottles obtained exclusively from PV
CVC episode	Episode consisting of a group of BC bottles with at least one sample from a CVC. Any peripheral vein samples part of the same episode is considered part of the CVC episode
Solitary episode	Episode consisting of a single BC set or of a single BC bottle
Contaminant organisms panel	CoNS (excluding *S. lugdunensis*), *Micrococcus* spp., *Cutibacterium acnes*, *Corynebacterium* spp. (excluding *C. jeikeium and C. striatum*), *Bacillus* spp., *Lactobacillus* spp., *Aerococcus* spp.
Restrictive contamination criteria	Non-solitary PV episode with an isolated organism belonging to the contaminant organisms panel found in only one BC bottle
Contaminations after manual review	Manual definition by an operator of adjunctive contaminated events. The system gives the opportunity of reviewing non-solitary episodes with an organism belonging to the contaminant organisms panel found in > 1 BC bottles (any number of bottles) and classifying them as contaminated or not

*BC* blood culture, *CoNS* coagulase-negative staphylococci, *CVC* central venous catheter, *PV* peripheral vein

BD DREAM™ BSI automatically elaborates the following KPIs: KPI (1) mean blood volume (per bottle, stratified per center, per area and per hospital service); KPI (2) rate of solitary episodes over the total of episodes; KPI (3) rate of episodes where a CVC episode does not include a set from peripheral vein (PV) over the total of CVC episodes; KPI 4a) rate of contaminations (with “restrictive contamination criteria”), over the total of non-solitary PV episodes.

Adjunctively to the standard categorization of contamination episodes, the tool offers the opportunity for an expert evaluation of contamination, allowing the visualization of the BC bottles in the context of the episode. The user can operate a manual editing for definition of “contaminations after manual review” selecting among those with an organism belonging to the contaminant organisms panel found in > 1 BC bottles. In this paper, the criterium used for the definition of “contamination after manual review” was: positivity for the same contaminant organism in two BC bottles belonging to the same BC set (where identification of BC sets was possible in the context of a MSS) over all the bottles collected during the BSI episode. Therefore, it is possible to calculate the KPI 4b representing the rate of contaminations (“restrictive contamination criteria” plus “contaminations after manual review”), over the total of non-solitary PV episodes.

### Involved centers and areas

**Fig. 2:**
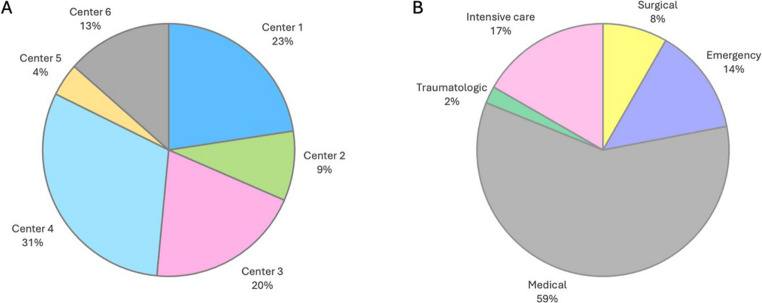
Percentages of BC bottles included in the study, per center (**A**) and per area (**B**)

A multi-center, real-life, implementation of the BD DREAM™ BSI was performed at 6 hospitals (5 tertiary hospitals and 1 specialized hospital) distributed across the Italian territory. Data were analyzed at the hospital, hospitalization area and hospital service level. Hospital services were stratified in “areas” according to the classification proposed by the Italian Ministry of Health [15]. Percentage of BC bottles per center and per area over the total of included bottles are shown in Fig. 2.

The study period was 1 st January − 31 st December 2023 and only BC bottles (BD BACTEC™ Plus Aerobic/Anaerobic medium, BECTON DICKINSON and Company) from adult patients (> = 18 years old), analyzed using BD BACTEC™ FX instrumentation (BECTON DICKINSON and Company), were included.

## Results

Overall, the total number of BC bottles analyzed with the BD DREAM™ BSI was 234,978 (118,399 BC sets). A total of 55,819 episodes from 26,559 patients (mean: 2.1 episodes/patient) were included. The number of BC sets ordered per 1,000 patient days was 132.3, which is consistent with the recommended 100 to 200 BC sets per 1,000 patient days [16].

Over the total of episodes, 11,959 (21.4%) were CVC episodes while the remaining included BC bottles obtained from PV only. The majority of episodes were from the medical area (59.1%) with a high rate of CVC episodes in the intensive care unit (ICU) area.

The positive blood culture rate varied among centers from 13.2 to 24.2% (mean 20.6%) (Table 2).

### KPI 1

At included centers, the mean blood volume per bottle was 6.45 mL (range 5.65–7.37 mL) and the mean blood volume per episode was 27.15 mL (range 18.55–36.07 mL). The minimum and maximum values found in participating hospital services (mean value per hospital service, shown per center and per area) is available in Supplementary Table 1.

### KPI 2

Overall, the mean number of bottles per episode was 4.21, ranging from 2.87 to 5.22 (stratified per center), and the overall rate of solitary BC was 21.8%. A wide variability was recorded among centers regarding KPI 2 (range stratified per center 3.2–62.9%). The lowest mean number of BC bottles/episode was found in the Emergency area (3.76), while the lowest mean volume of blood per BC bottle and per episode was in the medical area (5.75 mL and 24.73 mL, respectively). The highest rate of solitary BCs was found in the Surgical area (23.9%).

### KPI 3

The overall percentage of CVC episodes without BC bottles obtained from PV was 14.9% (range stratified per center 10.2–39.0%). In the ICU, the percentage of CVC episodes without BC bottles obtained from PV was 9.1% (Table 2).

**Table 2:** KPIs determination for the included centers and for areas

	*N*. patients	*N*. BC bottles	*N*. episodes	Mean BC bottles/episode	*N*. CVC episodes (%)	Positive episodes (%)	Mean blood volume/BC bottle (mL) KPI 1	Mean blood volume/episode (mL)	*N*. Solitary BCs	Rate of solitary BCs (%); KPI 2	*N*. unpaired CVC episodes	Rate of unpaired CVC episodes (%); KPI 3
Center n.												
Center 1	5,291	53,215	10,197	5.22	2,559 (25)	23.0	6.91	36.07	331	3.2	262	10.2
Center 2	3,121	20,828	6,406	3.25	561 (9)	24.2	5.71	18.55	3,813	59.5	219	39.0
Center 3	4,902	47,011	10,770	4.36	2,188 (20)	17.2	5.65	24.66	2,674	24.8	340	15.5
Center 4	7,512	72,439	17,265	4.2	4,008 (23)	20.8	6.94	29.12	1,782	10.3	487	12.2
Center 5	1,686	9,771	3,401	2.87	781 (23)	13.2	7.37	21.18	2,142	62.9	188	24.1
Center 6	4,047	31,714	7,780	4.08	1,862 (24)	22.4	5.87	23.94	1,407	18.1	281	15.1
Total	26,559	234,978	55,819	4.21	11,959 (21.4)	20.6	6.45	27.15	12,149	21.8	1,777	14.9
Area												
Surgical		19,619	4,820	4.07	1,189 (25)	19.2	6.96	28.33	1,154	23.9	181	15.2
Emergency		32,267	8,584	3.76	277 (3)	32.3	6.59	24.75	1,367	15.9	43	15.5
Medical		138,848	32,293	4.30	6,667 (21)	16.8	5.75	24.73	7,383	22.9	1,193	17.9
Traumatology		5,101	1,151	4.43	125 (11)	16.2	6.95	30.80	160	13.9	24	19.2
ICU		39,143	8,971	4.36	3,701 (41)	24.3	8.55	37.29	2,085	23.2	336	9.1
Total		234,978	55,819	4.21	11,959 (21)	20.6	6.45	27.15	12,149	21.8	1,777	14.9

*BC* blood culture, *CVC* central venous catheter, *ICU* intensive care unit

**Fig. 3:**
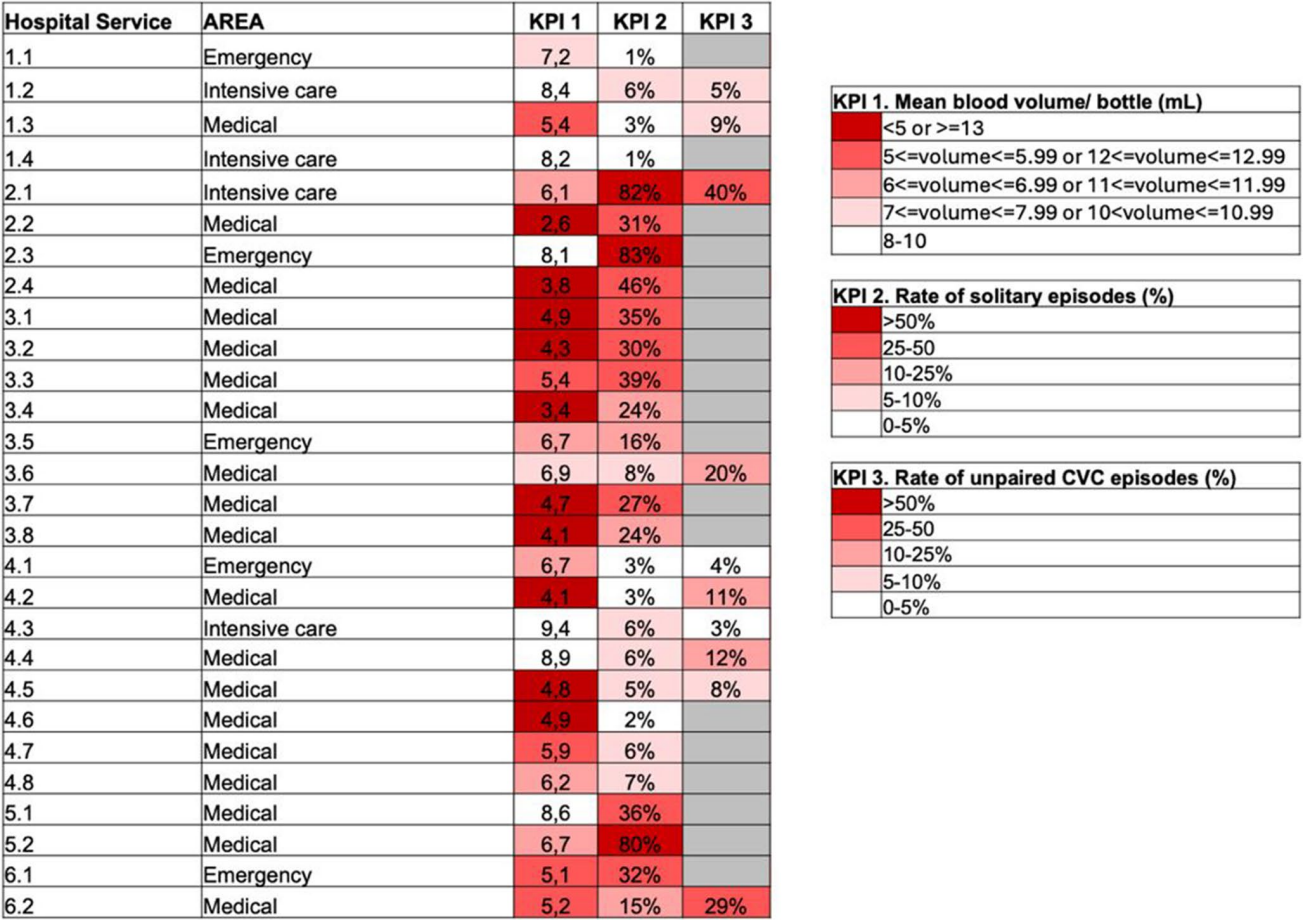
Details of the monitored KPIs stratified by hospital service. Critical KPIs values are represented with high intensity red color. Only hospital services with a representative number of episodes are shown. The following thresholds were adopted: >500 episodes for KPIs 1 and 2; >75 CVC episodes for KPI 3. Gray boxes were used for KPI/hospital service combinations below the threshold

The BD DREAM™ BSI enables the visualization of KPIs at the hospital service level, also. The visualization of KPIs stratified at this level gives rapid feedback useful for identification of wards with more critical performance (Fig. 3).

### KPI 4

**Fig. 4:**
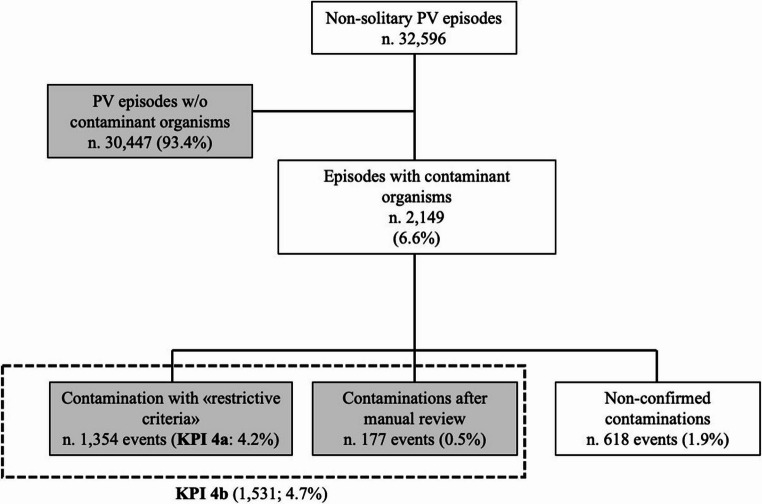
Diagram resuming the process categorization of contaminations with “restrictive contamination criteria” and “contaminations after manual revision”. PV: peripheral vein

Among the 43,346 PV episodes, 10,750 were solitary. Therefore, the system evaluated the contamination rate considering the remaining 32,596 non-solitary episodes. Among these, an organism included in the “contaminant organisms panel” was found in 2,149 episodes (6.6%). Of these episodes, 1,354 also met the “restrictive contamination criteria” (KPI 4a: 4.2%). After manual revision, 177 “contaminations after manual review” were recovered (177/32,592 episodes: 0.5%) (Table 3; Fig. 4). Consequently, the contamination rate after manual review raised to 4.7% (KPI 4b). The highest levels (for both KPI4a and KPI 4b) of contamination were observed in the Emergency area and in the ICU area (Table 3). The ranking of species found in episodes matching “restrictive contamination criteria” or “contaminations after manual review”, in decreasing frequency order, was: coagulase-negative staphylococci (CoNS) 91%; *Corynebacterium* spp. 4%; *Micrococcus* spp. 4%; *Cutibacterium acnes* 2%; others (*Bacillus* spp., *Lactobacillus* spp., *Aerococcus* spp.) 1%.

**Table 3:** Contamination rates for individual participant hospital and for areas

Contamination rate	Center *N*.						Total	Area					Total
	1	2	3	4	5	6		Surgical	Emergency	Medical	Traumatology	ICU	
KPI 4a (episodes, %)	310 (4.2)	124 (5.9)	190 (3.2)	511 (4.3)	21 (2.1)	198 (4.3)	1,354 (4.2)	67 (2.8)	419 (6.0)	589 (3.2)	34 (4.1)	245 (6.6)	1,354 (4.2)
KPI 4b (manual review) (episodes, %)	354 (4.8)	127 (6.0)	234 (4.0)	586 (5.0)	22 (2.2)	208 (4.5)	1.531 (4.7)	80 (3.3)	481 (6.9)	645 (3.5)	37 (4.4)	288 (7.8)	1,531 (4.7)

*ICU* intensive care unit

## Discussion

Monitoring the quality of key steps in complex processes is essential. Therefore, several key performance indicators have been identified and are increasingly monitored within healthcare institutions as part of quality assurance activities. Several KPIs have been proposed for monitoring the BSIs diagnostic pathway [1, 13, 17–21].

However, monitoring KPIs is a demanding activity, often out of the capabilities of most of Clinical Microbiology Laboratories. A previous work, performed in 2016, showed that the majority of Italian Microbiology Laboratories monitor BC KPIs in a non-systematic manner [21]. The increasing availability of informatic tools for the management of laboratory data offers increasing opportunities for KPIs automated monitoring.

In this paper, we show the functioning of a new, proprietary, digital remote extraction analysis and monitoring software that offers support in the monitoring of the BC process automatically elaborating customizable reports stratified at the hospital, area and ward level. Adopting the proposed approach, it is possible to rapidly and automatically extract data in a reliable manner and at planned intervals (e.g., monthly at the single ward, department or hospital level).

A “real life” evaluation of the tool functioning was performed at Italian hospitals obtaining an updated overview of BC KPIs in our setting, using a large set of data (more than 230,000 BC bottles).

Overall, the mean blood volume per bottle was 6.45 mL/bottle with none of the involved centers reaching the lower limit of the standard (8.0 mL). Similarly, the mean blood volume per episode remained below the standard (30–40 mL, according to Italian guidelines) in 5 out of 6 centers. Interestingly, considering admission areas, both values were acceptable in the ICU setting only, probably because in ICU patients, drawing blood from the CVC or artery could be easier than from the PV. It has been demonstrated that an insufficient blood volume per episode is statistically associated with an increased likelihood of false negative results, with negative effects on patient therapeutic management and outcome [22].

In a previous work, 26–36% of bottles collected in 5 Belgian hospitals were underfilled. However, in that experience, only 4 of the 5 participating hospitals collected data from a two weeks period, while the tool described in our work enables continuous and detailed monitoring of the KPIs [23]. For instance, adopting the proposed system, it is possible to organize a prompt and easy feedback to clinical services (e.g. a standard report generated by BD DREAM™ can be sent to wards monthly).

The wide implementation of the described tool could represent a significant advancement in the capability of monitoring BC bottles filling level of the Italian clinical microbiology laboratories that, historically, declared to be able to monitor these KPIs in less than 50% of cases [21].

Regarding the rate of solitary episodes, the diversity among centers was extremely high. The overall rate of solitary episodes was 21.8% (starkly above the target of 10%) [13], but centers with excellent data (< 4% of solitary episodes) coexisted with centers with unacceptable performance (> 60%). The setting with the highest value was the Surgical area. Surprisingly, the rate of solitary episodes was lower at the Emergency department. The diversity of data reported from centers likely reflects differences existing in personnel training programs and, probably, also different degrees of understaffing among centers. Solitary BC negatively impacts on BC sensitivity with the appropriate number of bottles per episode being from four to eight [13]. In a single-center study, based on 629 monomicrobial BSI episodes, it has been demonstrated that increasing the number of BC bottles per episode from two to four or six increases the positivity rate of 15% and 25%, respectively [24]. In our multicenter, larger experience the positivity rate (excluding contaminated BCs) among solitary episodes was 11.75% versus a positivity rate in non-solitary episodes of 15.68% (data not shown).

The most widely adopted criteria for the definition of intravascular catheter-related infection is the differential time to positivity (DTP) approach [25]. With this approach, episodes with BCs obtained from intravascular catheter only (most frequently a CVC) are not classifiable as catheter-related versus non-catheter-related. In our study, the overall rate of CVC episodes without BC bottles obtained from PV was 14.9%. To our best knowledge, no specific data on this KPI have been published before. Interestingly, the centers with higher rates were also those where other KPIs (BC filling volume and rate of solitary episodes) were more critical.

Contamination of BCs is defined as the presence of a microorganism that has been introduced into the culture during either specimen collection or processing and that is not pathogenic for the patient. Contaminations have negative effects on patient outcome and the whole system. In fact, patients with contaminated BCs need more consultations, have longer length of stay and receive more unnecessary antibiotics [26]– [27].

The criteria adopted for the definition of contaminated BC are controversial, with the most widely accepted being those defined by the Clinical Laboratory Standards Institute (CLSI) [28].

Briefly, CLSI considers a BC contamination as the identification of an organism of a defined panel list in only one of a series of BCs (either for positivity of one or both bottles of a single BC set). A series of BCs is defined as the group of one or more specimens collected within a 24 h period [28]. However, this definition could underperform in institutions adopting the “single sampling strategy” (SSS) [10]. In this case, the distinction in various sets does not apply since all BC bottles are inoculated within the same sampling procedure. In our study, we were not able to discriminate between episodes associated with BCs obtained with the SSS versus the MSS. SSS has been widely adopted in Italy to reduce the workload and the rate of solitary episodes [29]– [30]. For this reason, we configured the system to automatically provide a contamination rate based on “restrictive criteria” (presence of an organism within the “contamination organisms panel” in just one BC bottle of a non-solitary episode; acceptable for both MSS and SSS) and to give to the operator the opportunity of manual reviewing adjunctive suspect contaminated episodes. This is particularly useful for institutions adopting MSS. In this situation the system can be manually set to obtain a contamination rate compliant with CLSI criteria. In our set of data, the manual revision led to recovery of just 177 contaminated episodes (0.5%). In addition, our work provides a ranking of organisms most frequently found in confirmed contamination episodes. Remarkably, we found that more than 90% of contaminants were CoNS. This percentage is higher than that reported previously [31].

Comparing to previous experiences of digital monitoring of KPIs (using BacT/Alert Virtuo, bioMérieux INC. Marcy l’Etoile, France) [32], our approach display an higher level of automation and the process is entirely completed by a proprietary software, without necessity of third party tools utilization (e.g. Microsoft Excel).

Our approach has some limitations also. In fact, while the BD DREAM™ tool significantly advances the automation of KPIs monitoring, its effectiveness relies on the quality of the underlying data (for example the determination of the contamination rate after manual review is affected by the correct identification of different sets of BCs collected in the context of the same episode). Any discrepancies in data entry or extraction processes could affect the accuracy of the results. Furthermore, the manual review process used to confirm contamination could introduce bias or subjectivity. By contrast, the adoption of the automated tool for contamination rate using “restrictive criteria” could lead to underestimate the real contamination rate.

Finally, a further limitation was that we were unable to record the time between blood sampling and incubation of BCs in continuous monitoring systems. This could represent a further improvement of the system in the future.

In this work, we demonstrate that full automation of the BCs KPIs monitoring is feasible by adopting appropriate digital tools. However, we did not perform an evaluation of the impact of the implementation of the proposed approach. This can be considered as a limitation of the present study. This aspect will be the subject of other studies in the future.

## Supplementary Information

Below is the link to the electronic supplementary material.

Supplementary Table 1. Minumum and maximum KPI 1, 2 and 3 values stratified per center and per area. (10.1 KB)

## Data Availability

The authors confirm that all supporting data have been provided within the article.

## Appendix

GLIPaC (Gruppo di Lavoro per le Infezioni nel Paziente Critico) Working Group of “Associazione Microbiologi Clinici Italiani” (AMCLI ETS): Fabio Arena, Giulia Menchinelli, Marta Argentieri, Paola Bernaschi, Carla Fontana, Massimo Giusti, Flora Marzia Liotti, Giovanni Lorenzin, Antonella Mencacci, Cinzia Peronace, Mario Rassu, Teresa Spanu, Bruno Viaggi, Teresa Lopizzo.
